# Identification of potential target genes for the tomato fruit-ripening regulator RIN by chromatin immunoprecipitation

**DOI:** 10.1186/1471-2229-11-26

**Published:** 2011-01-30

**Authors:** Masaki Fujisawa, Toshitsugu Nakano, Yasuhiro Ito

**Affiliations:** 1 National Food Research Institute, 2-1-12 Kannondai, Tsukuba, Ibaraki 305-8642, Japan

## Abstract

**Background:**

During ripening, climacteric fruits increase their ethylene level and subsequently undergo various physiological changes, such as softening, pigmentation and development of aroma and flavor. These changes occur simultaneously and are caused by the highly synchronized expression of numerous genes at the onset of ripening. In tomatoes, the MADS-box transcription factor RIN has been regarded as a key regulator responsible for the onset of ripening by acting upstream of both ethylene- and non-ethylene-mediated controls. However, except for *LeACS2*, direct targets of RIN have not been clarified, and little is known about the transcriptional cascade for ripening.

**Results:**

Using immunoprecipitated (IPed) DNA fragments recovered by chromatin immunoprecipitation (ChIP) with anti-RIN antibody from ripening tomato fruit, we analyzed potential binding sites for RIN (CArG-box sites) in the promoters of representative ripening-induced genes by quantitative PCR. Results revealed nearly a 5- to 20-fold enrichment of CArG boxes in the promoters of *LeACS2*, *LeACS4*, *PG*, *TBG4*, *LeEXP1*, and *LeMAN4 *and of *RIN *itself, indicating direct interaction of RIN with their promoters *in vivo*. Moreover, sequence analysis and genome mapping of 51 cloned IPed DNAs revealed potential RIN binding sites. Quantitative PCR revealed that four of the potential binding sites were enriched 4- to 17-fold in the IPed DNA pools compared with the controls, indicating direct interaction of RIN with these sites *in vivo*. Near one of the four CArG boxes we found a gene encoding a protein similar to thioredoxin y1. An increase in the transcript level of this gene was observed with ripening in normal fruit but not in the *rin *mutant, suggesting that RIN possibly induces its expression.

**Conclusions:**

The presented results suggest that RIN controls fruit softening and ethylene production by the direct transcriptional regulation of cell-wall-modifying genes and ethylene biosynthesis genes during ripening. Moreover, the binding of RIN to its own promoter suggests the presence of autoregulation for *RIN *expression. ChIP-based analyses identified a novel RIN-binding CArG-box site that harbors a gene associated with *RIN *expression in its flanking region. These findings clarify the crucial role of RIN in the transcriptional regulation of ripening initiation and progression.

## Background

Ripening processes of many kinds of fruit involve various biochemical and physiological changes, such as softening, enrichment of pigments, organic acids and nutrients (*e.g.*, vitamins and sugars), and development of aroma and flavor. These changes make fruits attractive for the human diet. For climacteric fruits, autocatalytic ethylene production and an increase in respiration occur during ripening, and ethylene has been well characterized as necessary for the coordination and completion of ripening [1]. At the onset of ripening, expression patterns of numerous genes involved in these ripening-associated phenomena are upregulated in a highly synchronized fashion, indicating that ripening is controlled by a highly systematic and sophisticated transcriptional mechanism. Therefore, much attention has been paid to how fruit ripening is regulated because ripening regulation is not only of agricultural importance but also of scientific interest in terms of the regulation of biological developmental processes. However, a substantial portion of the genetic regulatory mechanism controlling the process remains unclear.

The tomato (*Solanum lycopersicum*) is the most advantageous model plant for the study of fruit ripening due to its climacteric ripening nature, availability of the genome information and many suggestive mutations concerned in ripening [2,3]. Among the ripening mutations, *ripening inhibitor *(*rin*) is a well-characterized mutation that inhibits such characteristic phenomena observed during ripening as lycopene accumulation and softening, resulting in non-ripe fruit [4]. The *rin *mutation also inhibits autocatalytic ethylene production during ripening; thus, the wild-type gene on the *rin *locus has been regarded as a regulator responsible for the onset of ripening by acting upstream of both ethylene- and non-ethylene-mediated ripening control. The *rin *locus has been isolated and found to encode two MADS-box transcription factors, RIN and MC (Macrocalyx), and RIN is apparently responsible for the regulation of fruit ripening [5]. Molecular characterizations have revealed that *RIN *is expressed during ripening specifically, that the gene product exhibits transactivation activity and that RIN has the ability to bind to the specific DNA sequences known as C-(A/T)-rich-G (CArG) box, which is a typical binding sequence for MADS-box proteins [6].

To identify genes associated with ripening phenomena, the genes whose expressions are affected by the *rin *mutation have been extensively investigated. In ethylene biosynthesis and signaling, the transcription levels of the genes encoding 1-aminocyclopropane-1-carboxylic acid (ACC) synthase 2 (LeACS2), ACC synthase 4 (LeACS4), ACC oxidase 1 (LeACO1) and ethylene receptor protein 3 [ETR3; synonymous with NEVER RIPE (NR)] increase dramatically during ripening, but their transcriptions are inhibited by the *rin *mutation, indicating that these genes are responsible for the elevation of ethylene level and for ethylene signaling during ripening [7-9]. The *rin *mutation also inhibits upregulation of the genes involved in cell-wall modifications, such as *Polygalacturonase *(*PG*), *β-Galactosidase 4 *(*TBG4*), *Endo-(1,4)-β-mannanase 4 *(*LeMAN4*) and *α-Expansin 1 *(*LeEXP1*), all of which are assumed to be concerned with softening and the shelf life of fruit [10-19]. Ripening-associated upregulation of the gene for phytoene synthase 1 (PSY1), which is a rate-limiting enzyme for carotenoid production including lycopene in ripening tomatoes, and the gene for β-fructofuranosidase [also called invertase (INV)], which catalyzes hydrolysis of sucrose in ripening fruit, is also affected by the *rin *mutation [20-22]. In addition, a DNA microarray assay revealed that a large number of genes upregulated during ripening were suppressed by the *rin *mutation [23]. These results apparently indicate that the RIN protein is a transcriptional regulator triggering the onset of ripening by inducing the expression of these ripening-associated genes. Three pathways for the transcriptional regulation of ripening-associated genes by RIN are possible. The first pathway is that RIN binds to the promoter of target genes and directly regulates their expression. The second is that RIN induces ethylene production at the onset of ripening and ethylene-induced genes are consequently transcribed. The third is that RIN induces some transcription factors directly, and subsequently the transcription factors induce the ripening-specific gene expressions. It is expected that these three pathways act in parallel and regulate the expression of the large number of ripening-associated genes, although the elements of these transcriptional cascades remain largely unknown except that RIN binds to the promoter of *LeACS2 *[6].

To learn more about these pathways, we have developed ChIP-based analyses for fruit ripening. ChIP, a technique to collect target DNA sequences of a protein of interest as protein-DNA complexes (chromatin) with an antibody for the protein, is a powerful tool used to ascertain interactions of transcription factors with DNA *in vivo *[24]. Comprehensive ChIP-based approaches have been used for identifying potential target genes of a few floral homeotic MADS-box transcription factors in plants [25-27] but have not been applied for analysis of fruit ripening to date. Here, we report identification of the *in vivo *RIN-binding sites in the putative promoters of several ripening-induced genes through ChIP-based analyses using ripening tomato fruit. In addition, we screened novel RIN-binding sites containing CArG boxes and found a candidate gene possibly regulated by RIN. The results offer insights into the characteristics of RIN in the transcriptional regulation at the onset of ethylene production and cell-wall modifications, and in the autoregulation of *RIN *itself during ripening.

## Results

### *In silico *search of CArG boxes in the promoters of ripening-induced genes

**Figure 1:**
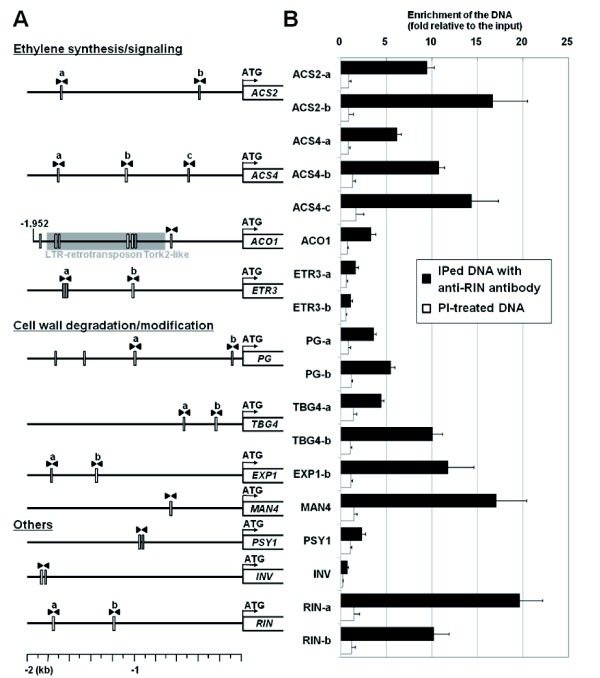
**CArG-box sites in the ripening-associated gene promoters and their enrichment in ChIP-DNA**. (A) Position of CArG-box sites (indicated by thin rectangles) found in the 2-kb potential promoter regions of the ripening-induced genes. A pair of primers specific to each site is indicated by pairs of filled arrowheads. When two or more sites exist in the same promoter, they are distinguished by the lower-case letters (a, b) above them. (B) Enrichment test of the CArG boxes. Bars represent the relative DNA amount of CArG boxes in IPed DNA recovered using either anti-RIN antibody or pre-immune serum (PI) to those in the total input chromatin DNA. (Note that the result of EXP1-a is omitted due to inadequate amplification efficiency in the real-time PCR analysis.) Data are the means from three independently prepared IPed DNAs. Error bars indicate standard error of each mean.

The *rin *mutant lacks expression of *LeACS2, LeACS4*, *TBG4*, *LeEXP1, LeMAN4 *and *PSY1*, and shows decreased expression levels of *LeACO1*, *ETR3*, *PG *and *INV*, while these genes are highly upregulated in the wild-type fruit [8,13,28,29], indicating that all of these genes are regulated directly or indirectly by RIN. To detect potential RIN-binding sequences, a possible CArG-box motif [C(C/T)(A/T)_6_(A/G)G] [6] was searched against the promoters of these genes (~2 kb). The motif includes three groups of CArG-box sequences: SRF-like [canonical CArG-box, CC(A/T)_6_GG] [30], MEF2-like [CTA(A/T)_4_TAG] [31], and intermediate [CC(A/T)_6_AG] [6,32]. A motif search revealed that all the genes except *LeACS4 *and *LeEXP1 *have at least one typical CArG-box sequence in their promoters (Table 1 and Figure 1A). Instead, the promoter of *LeEXP1 *was found to contain no typical RIN target motif, but rather two atypical CArG-box sequences, CATTTATATG and CAATTTAAAG (underlines indicate atypical bases; Table 1 and Figure 1A). The promoter of *LeACS4 *was also found to carry no typical but three atypical sequences of CAAATATAAG, CAATTTTAAG and CTAGTTAAAG (underlines indicate atypical bases; Table 1 and Figure 1A). We also further analyzed these atypical CArG-box sequences in the *LeEXP1 *and *LeACS4 *promoters as well as analyzing the possible CArG boxes.

**Table 1:** CArG-box sequences found in the promoters of ripening-induced genes.

Site	**CArG-box and its flanking sequences (5' to 3')**^**1)**^	**Motif **^**2)**^	**CArG-box position (bps)**^**3)**^
ACS2-a^4)^	AGCTATT-CTAAAAAAAG-TATCACATA^5^)	Possible	X59139 (1,365 - 1,374) (+)
ACS2-b	AAATGCAC-CCTAAATTAG-TCAAATAT^5^)	Possible	X59139 (2,654 - 2,663) (+)
ACS4-a	ATCAAACA-CAAATATAAG-TTTGGAAC^5^)	Atypical	M88487 (567 - 576) (+)
ACS4-b	ATTAAACA-CAATTTTAAG-AAACTTTT^5^)	Atypical	M88487 (1,201 - 1,210) (+)
ACS4-c	TGAAATAT-CTAGTTAAAG-ATATGTAC^5^)	Atypical	M88487 (1,802 - 1,811) (+)
ACO1	GGTTGAAT-CTATAAAAAG-AAAAATAT	Possible	X58273 (1,285 - 1,294) (+)
ETR3-a	GGAGAAAT-CCTATAATAG-GGCAAACA	Intermediate	AY600437 (3,121 - 3,130) (+)
	GAGAAATC-CTATAATAGG-GCAAACAC	Possible	AY600437 (3,122 - 3,131) (+)
	GGCAAACA-CCAAAAATAG-CTTGGAGT	Intermediate	AY600437 (3,139 - 3,148) (+)
ETR3-b	AAATTTCA-CTTAATATGG-ACTAGAGA	Possible	AY600437 (3,745 - 3,754) (+)
PG-a	GCACCAAT-CTAATTTAGG-TTGAGCCG	Possible	scaffold01076 (1,534,540 - 1,534,549) (-)
PG-b	CTTAAAAT-CTATAAATAG-ACAAACCC	MEF2-like	scaffold01076 (1,533,632 - 1,533,641) (-)
TBG4-a	TATATGCT-CTATTTTTGG-ACGGCAGG^5^)	Possible	scaffold00061 (457,025 - 457,034) (+)
TBG4-b	TTTGGGCC-CCATTTAAGG-GATTGGGC^5^)	SRF-like	scaffold00061 (457,311 - 457,320) (+)
EXP1-a	TTATTTTA-CATTTATATG-TTATTATT	Atypical	scaffold00114 (3,118,161 - 3,118,170) (-)
EXP1-b	TGATGCTT-CAATTTAAAG-AAAATAAA^5^)	Atypical	scaffold00114 (3,117,729 - 3,117,738) (-)
MAN4	TTTCTTTT-CCATTTATAG-AAAAACCA^5^)	Intermediate	scaffold01157 (9,316,653 - 9,316,662) (-)
PSY1	TATGTGTA-CCAAAATTAG-AAAATCAG	Intermediate	scaffold00066 (364,464 - 364,473) (+)
	CTTGTTGA-CTAAATATAG-AATGCATC	MEF2-like	scaffold00066 (364,504 - 364,513) (+)
INV	TTATGATA-CTTAATATGG-TAATCTTT	Possible	Z12027 (1,634 - 1,643) (+)
	TTCTCACT-CTATAAATAG-AGTTGTTC	MEF2-like	Z12027 (1,667 - 1,676) (+)
RIN-a	GTTGCACT-CTAAAAAAAG-TTAAAAGG^5^)	Possible	scaffold00243 (210,835 - 210,844) (-)
RIN-b	ACAAAGAA-CCATTAAAAG-GTTAAAAA^5^)	Intermediate	scaffold00243 (210,262 - 210,271) (-)

1) CArG-box sequences are underlined.

2) CArG-box sequences in the exhibited strand were grouped into motifs. Groups of the motif sequences are referenced in the text.

3) Scaffolds followed by Arabic numerals indicate that the sequences originate from WGS data. Symbols + and - in parentheses indicate that the CArG boxes displayed are presented in either the exhibited (+) or complementary (-) strands.

4) Site was previously described [6].

5) Sequences were used for *in vitro *gel retardation assays.

### Binding of RIN to the CArG boxes in the ripening-induced gene promoters *in vivo *and *in vitro*

To distinguish the genes directly regulated by RIN, we applied the ChIP assay. Chromatin was prepared from ripening tomato fruits harvested at the pink coloring stage and was then immunoprecipitated with the anti-RIN antibody. The resulting immunoprecipitated DNAs (IPed DNAs) were assayed by quantitative-PCR analyses (qChIP-PCR) for the regions containing the putative RIN-binding sites described above, and then enrichment levels of the regions were evaluated. The qChIP-PCRs were carried out with a few considerations as follows: (1) when two or more CArG-box sequences were closely located (within ~150 bp region of each other), they were treated as one site and tested together with a pair of primers designed to include them both (Table 1, Figure 1A and Additional File 1); (2) in the case of *PG*, the two closest sites to the protein-coding region were tested (Figure 1A); (3) the five CArG-box sites found between the closest and the farthest sites in the promoter region (1,952 bp) of *LeACO1 *were excluded from the test because they were considered to be similar to an LTR-type retrotransposon (Tork2-like; Figure 1A) [33] whose replicates are dispersed throughout the genome, preventing site-specific amplification, and because the farthest site (at 1.8-kb upstream) from the protein-coding region of *LeACO1 *could not be examined by primer pairs of reasonable length.

The result of qChIP-PCR revealed significant enrichment of all the CArG boxes in the IPed DNA pools relative to the total chromatin DNA pools (the input DNA) (Figure 1B) except INV (0.7-fold) and EXP1-a (the enrichment of which failed to be monitored due to unstable PCR amplification). In particular, ACS2-a, ACS2-b, ACS4-b, ACS4-c, TBG4-b, EXP1-b, MAN4, RIN-a and RIN-b were extremely enriched to 9.5-, 16.7-, 10.8-, 14.4-, 10.1-, 11.8-, 17.1-, 19.6- and 10.2-fold, respectively (Figure 1B). Furthermore, ACS4-a, PG-b and TBG4-a were moderately enriched to 6.2-, 5.5- and 4.5-fold, respectively (Figure 1B). These enrichments were not observed in the immunoprecipitated DNA prepared with the pre-immune serum (PI-treated DNA pools) (0.2 to 1.7-fold; Figure 1B), indicating specific binding of RIN to these 12 CArG boxes *in vivo*. Other CArG boxes (ACO1, ETR3-a and -b, PG-a and PSY1) showed relatively lower enrichment (3.3-, 1.6-, 1.1-, 3.6-, 2.3-fold, respectively).

**Figure 2:**
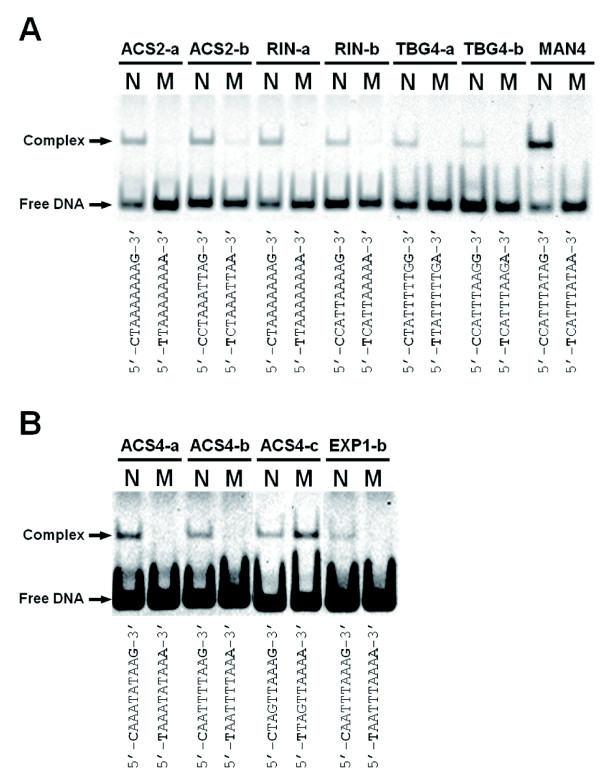
**Gel retardation assay of CArG boxes in the ripening-induced gene promoters**. Gel retardation assay of possible CArG boxes (A) and atypical CArG boxes (B) found in the promoters of the ripening-induced genes. DNA fragments of putative RIN-binding sites that contain a normal CArG-box sequence and flanking regions shown in Table 1 were reacted with the RIN-MIK protein *in vitro *and electrophoresed (lanes N). DNA fragments with mutations within the CArG box were also examined (lanes M). The normal CArG-box sequences (10 bp) and their mutant sequences are displayed below the gel images. Nucleotides substituted between the normal and mutated sequences are indicated by bold letters. The same amount of DNA fragments was applied to each lane in A and B. The image of B was adjusted to higher contrast than that of A due to the low intensities of the retarded signals for the sequences examined in B.

To identify the RIN-binding sequences within the enriched regions, the binding of RIN to the CArG-box sequences in the promoters of *LeACS2*, *LeACS4*, *TBG4*, *LeEXP1*, *LeMAN4 *and *RIN *were examined by *in vitro *gel retardation assay. Results showed that DNA fragments containing the CArG boxes were retarded in all the sites for these genes due to binding to the RIN protein (Figures 2A and 2B), although the signal intensity for atypical CArG-box sequences that include the three *LeACS4 *sites and EXP1-b appeared to be lower than that of the typical sequences, based on the ratio to the free DNA (Figures 2A and 2B). In contrast, by introducing mutations within the target sequences, the retardation was drastically inhibited in all target sequences except for ACS4-c (Figures 2A and 2B). These results indicate that RIN specifically recognizes the CArG-box sequences of the respective sites. In ACS4-c, the DNA fragment containing the mutated CArG box was retarded similarly to the normal sequence (Figure 2B), which is likely due to the unexpected generation of a sequence similar to the CArG box via base substitution (CTAAATATTT in the reverse strand; refer to the normal ACS4-c sequence in Table 1). This result indicates that RIN could bind to a wide spectrum of CArG-box sequences although *in vitro *RIN shows lower preference for the atypical motifs than for the typical CArG boxes.

### Genome mapping of DNA fragments recovered by ChIP and *in silico *detection of CArG-box sequences

To identify novel binding sites of RIN, we cloned the DNA fragments recovered by ChIP using the anti-RIN antibody. Of the IPed DNA clones, 51 were sequenced and mapped on independent genomic regions of the tomato (Table 2). The average length of the 51 cloned fragments was 380 bp (data not shown). To detect potential RIN-binding sequences, a possible CArG-box motif [C(C/T)(A/T)_6_(A/G)G] [6] was subsequently searched against the genomic regions. The search detected a total of 13 possible CArG boxes from 11 regions (Table 2). These boxes could be further categorized into four groups (Table 2). Because none of these regions has yet been reported to be bound by RIN, we considered them as novel potential RIN-binding sites and subjected them to further analyses.

**Table 2:** CArG-box sequences found in regions for which IPed DNAs were mapped.

CArG-box site	**CArG-box and its flanking sequences (5' to 3')**^**1)**^	**Motif (strand)**^**2)**^	**CArG-box position (bps)**^**3)**^
009F	CCTAAATA-CTATTATAAG-AATGATCA	Possible (+, -)	scaffold01172 (3,039,503 - 3,039,512)
016	GTACAGCA-CCAAAATTGG-CGACCACA	SRF-like (+, -)	scaffold01172 (4,647,499 - 4,647,508)
027R	AACTCTCC-CCTATATTGG-TGCTCAAT	SRF-like (+, -)	scaffold00008 (1,340,392 - 1,340,401)
042F	GATAGATC-CTAATTTTGG-TAAGTGAC	Possible (+), Intermediate (-)	scaffold00077 (3,770,305 - 3,770,314)
046F_1	CTTTTGGG-CTTAATTTAG-GGATTTAC	Possible (+, -)	scaffold00162 (1,604,081 - 1,604,090)
046F_2	ACATTTTT-CCATATTTAG-TACTAGAT	Intermediate (+), Possible (-)	scaffold00162 (1,604,514 - 1,604,523)
066F	ACTAGCAA-CTATTATAGG-GCCCTCCT	Possible (+), Intermediate (-)	scaffold00041 (5,908,092 - 5,908,101)
073F	AAAGTCCC-CTTTTTTTGG-AAAAATAC	Possible (+, -)	scaffold00885 (1,175,686 - 1,175,695)
090R	TATATTGT-CTATTATAGG-GGACGGTC	Possible (+), Intermediate (-)	scaffold00235 (679,848 - 679,857)
100_1	GCTGGATT-CTATTATAAG-GACATCAT	Possible (+, -)	scaffold00073 (9,132 - 9,141)
100_2	GCCAGATT-CCTATATTAG-CAGTATAG	Intermediate (+), Possible (-)	scaffold00073 (8,936 - 8,945)
128R	CTTCATAC-CTTAATTAAG-CAACCTTA	Possible (+, -)	scaffold00183 (1,735,586 - 1,735,595)
133R	AGAAAATG-CCATTTTTGG-AAGGAAGA	SRF-like (+, -)	scaffold00103 (1,409,234 - 1,409,243)

1) CArG-box sequences are underlined. Sequences displayed are the same strand as the WGS data (version 1.03) released by SGN. They were used for *in vitro *gel retardation assay.

2) Symbols + and - in parentheses indicate that the CArG-box sequences in the exhibited (+) or complementary (-) strands could be grouped into the motifs. Groups of the motif sequences are referenced in the text.

3) Positions on the WGS are displayed.

### Binding of RIN to the novel CArG-box sites *in vivo *and *in vitro*

**Figure 3:**
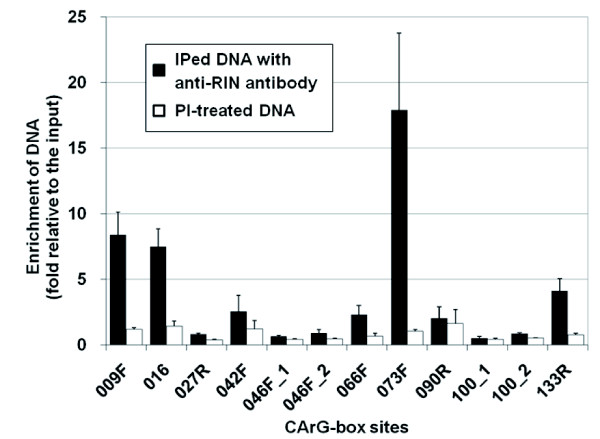
**Enrichment tests of the novel CArG boxes**. Bars represent the relative DNA amount of CArG boxes in IPed DNA recovered either with anti-RIN antibody or pre-immune serum (PI) to those in the total input chromatin DNA. (Note that the result for 128R is omitted due to the inadequate amplification efficiency in the real-time PCR analysis.) Data are the means from three independently prepared samples by ChIP with the anti-RIN antibody or the pre-immune serum. Error bars indicate standard error of each mean.

To examine the binding of RIN to the CArG-box sites within the cloned fragments, we monitored their enrichment levels in the IPed DNA pools by qChIP-PCR. Results showed that the DNA fragments of four sites, 009F, 016, 073F and 133R, were significantly enriched to 8.4-, 7.5-, 17.9- and 4.1-fold in the IPed DNA pools, respectively, compared with those in the input chromatin DNA. Such significant enrichment was not observed in the PI-treated DNA (only 0.4 to 1.4-fold; Figure 3), indicating that enrichment depends on the presence of the anti-RIN antibody, *i.e.*, these sites are specifically bound by RIN *in vivo*. Compared with these four sites, the other eight sites examined here showed relatively lower enrichment (0.5 to 2.6-fold; Figure 3).

**Figure 4:**
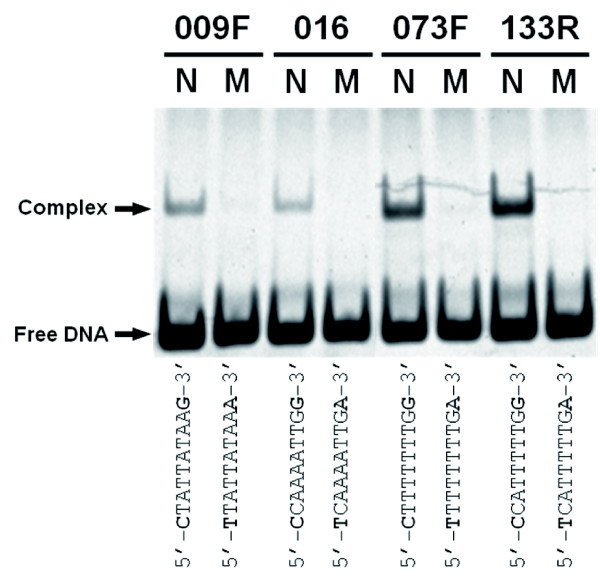
**Gel retardation assay of CArG boxes**. DNA fragments of the sites containing the normal (lane N) and mutated (lane M) CArG boxes were reacted with the RIN-MIK protein *in vitro *and electrophoresed.

The binding of RIN to the four sites was also examined by *in vitro *gel retardation assay. Results showed that the mobility of DNA fragments containing the normal CArG-box sequences was delayed in all the sites by binding to the RIN protein (Figure 4). This retardation was not detected at any site when mutation-introduced target sequences were used (Figure 4), indicating that RIN binds specifically to these CArG boxes.

### Genes in the flanking regions of the RIN-binding CArG boxes and their expression

**Figure 5:**
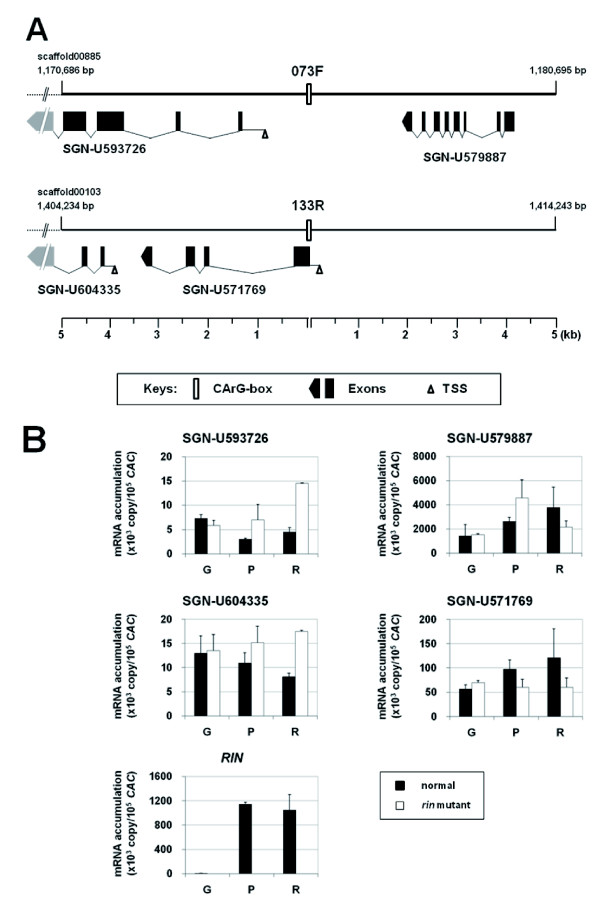
**Position, structure and expression pattern of predicted genes on flanking genomic regions of CArG boxes**. (A) Sequence positions of regions in the scaffolds from the WGS of tomato are indicated above the ends of the horizontal lines. Positions of the CArG boxes in the scaffolds are described in Table 1. All of the mapped ESTs were encoded on the complementary strand (in the right to left orientation). The exons mapped outside of the regions (broken lines) are shown in gray but are not to scale. Note that TSS for a gene corresponding to SGN-U579887 could not be predicted. The ruler below each diagram indicates the distance from the CArG boxes. (B) Expression analyses of the mapped ESTs. Lanes G, P and R represent the mature green, pink coloring and red ripe stages of tomato fruits, respectively. Data are the means from two independent experiments.

To detect potential target genes of RIN, we analyzed the 5-kb genomic regions flanking the four CArG boxes. A BLAST search of each flanking region of 073F and 133R using the SGN unigene set identified two potential genes corresponding to tomato ESTs, SGN-U579887 and SGN-U593726 for the former region, and SGN-U571769 and SGN-U604335 for the latter region (Figure 5A, Additional File 2). Gene predictions revealed that the CArG-box of 073F was located at the promoter region of the gene for SGN-U593726 [718 bp upstream of the predicted transcription start site (TSS)], while the CArG-box of 133R overlapped both the 5'-untranslated region (UTR) and the protein-coding sequence for SGN-U571769 (Figure 5A). By contrast, no EST was detected in the flanking 5-kb regions of 009F and 016.

If these four genes are under the transcriptional regulation of RIN, their expression pattern should be associated with that of RIN. To examine this, we analyzed their expression in normal and *rin *mutant tomato fruits together with the expression of the *RIN *gene. Similar to previously reported observations [5,29], the *RIN *mRNA level was extensively increased in normal fruit at the ripening stages [pink coloring (P) and red ripe (R)] compared with the pre-ripening stage [mature green (G)], but not in the *rin *mutant at the corresponding stages (Figure 5B). Among the genes found around the RIN-binding sites, SGN-U571769 showed an increased mRNA level with ripening in normal fruit, but no such increase was observed in the mutant fruit. This expression pattern seemed to coincide with that of *RIN *although SGN-U571769 was expressed also in the G stage to some extent and its level was relatively lower (~1/10) than that of *RIN *at the ripening stages (Figure 5B). Unexpectedly, however, other genes examined did not exhibit the expression pattern associated with *RIN *(Figure 5B).

## Discussion

### Selectivity of target sequences of RIN for tomato genome

Based on our analyses to identify direct target genes of RIN, we successfully identified a number of sites to which RIN binds in the genome of ripening fruit cells by screening the promoters of the genes that are highly upregulated during ripening and also within the DNA fragments prepared by the ChIP with the anti-RIN antibody. Results for the RIN target sites provide insight into how RIN selects target genes within the tomato genome.

Our previous *in vitro *assays demonstrated that RIN can bind to sequences of C(C/T)(A/T)_6_(A/G)G, and that the preferential binding sequence is 'CCA(A/T)(A/t)(A/T)ATAG' [6]. Here, we demonstrated that a number of the ripening-associated genes contain these typical binding sites in their promoters and that RIN actually binds to these sites. In contrast, however, the promoter of *LeACS4 *contains no typical RIN-binding sequences but does contain three atypical CArG-box sequences, CAAATATAAG (ACS4-a), CAATTTTAAG (ACS4-b) and CTAGTTAAAG (ACS4-c) (underlines indicate atypical bases). Similarly, the promoter of *LeEXP1 *also contains one atypical CArG-box sequence, CAATTTAAAG. ChIP assays demonstrated that these four atypical sites were enriched within the IPed DNA at a high level comparable to the sites within the promoters of *LeACS2 *and *LeMAN4 *that contain the typical binding sequences. This result indicates that *in vivo *RIN binds to a wider spectrum of CArG-box sequences within the genome than previously expected based on *in vitro *assay.

This ability of RIN to bind with a wide range of CArG-box sequences *in vivo *suggests that other factors might be necessary for RIN to target only the ripening stage-specific genes. The binding site selectivity of RIN might be affected by chromatin structure (*e.g.*, histone modification or DNA methylation) that controls the interaction of RIN with target DNAs in living cells. As another possibility, the selectivity of RIN might be increased by a tetramer- or higher-order multimer formation with other MADS-box proteins. For instance, in the 'quartet model' of floral organ specification, MADS-box proteins in floral organs form four combinations of tetramers that determine the identity of the different floral organs [34]. In this model, two dimers comprising the tetramer should recognize two different CArG-box sites, which confer higher selectivity of binding sites on transcription factor complexes [35]. In fact, Egea-Cortines et al. have proved that tetramer formation of MADS-box proteins dramatically increases the DNA-binding affinity [36]. In this study, we found that the *cis*-elements of *LeACS2, LeACS4, TBG4 *and *RIN *contain two CArG-box sites enriched at a significant level by ChIP using the anti-RIN antibody. These results suggest that RIN functions as a component of a tetramer that interacts with two binding sites of the target sites, just like the 'quartet model' transcription factor complexes. RIN possesses the ability to form a homodimer or heterodimers with other tomato MADS-box proteins belonging to the APETALA1/FRUITFULL subfamily (TM4, SLMBP7 and LeMADS1) and AG subfamily (TAGL1 and TAGL11) [6,37]. Because RIN belongs to the SEPALLATA (SEP) family among plant MADS-box group proteins [5,38], RIN might mediate interactions between other MADS-box proteins, similar to SEP proteins, which have been shown to mediate interactions between floral homeotic MADS-box proteins [39].

In this study, we identified novel RIN-binding sites from a screening of IPed DNA. At the regions flanking those sites, however, we found only one gene that is upregulated during ripening. Although we cannot exclude the possibility that the RINs binding to the other sites might regulate the genes located far from the sites (>5-kb), the bindings are likely not to activate any transcriptions. Similar observations were previously reported in the investigation of transcription factors of SEP3 through ChIP with Solexa sequencing (ChIP-SEQ), which showed that several percent of thousands of peaks are located far from protein-coding gene loci [27]. These facts mean that RIN-binding sites might not be restricted only to the promoter region of the RIN-inducible genes, but might include other genomic regions with CArG box-like sequences. If so, the binding of RIN at the *cis-*elements is necessary but not sufficient to induce the gene expressions required for fruit ripening, and certain factors are necessary for the specific transcriptional regulation by RIN. An assay using a yeast system revealed that RIN activates the transcriptional activity of an RNA polymerase that binds to the flanking genomic region [6], suggesting that the transactivation activity of RIN requires other transcriptional machinery at the RIN-binding sites. Higher-order multimer formation and other transcriptional machinery might allow RIN to induce specific gene expressions during ripening.

### Autoregulation of RIN and the promising direct target genes of RIN

Our analysis revealed that RIN binds to the CArG-box sites in its own promoter *in vivo*, strongly suggesting that RIN autoregulates its own expression. The existence of this autoregulatory mechanism can explain how the rapid increase in the mRNA level of *RIN *at the onset of ripening is controlled [5,29] (Figure 5B), *i.e.*, it is expected that autoregulation apparently controls the expression of *RIN *in a positive manner during ripening due to the transcriptional activation activity of RIN [6]. Autoregulation is frequently observed in plant floral homeotic MADS-box genes such as *DEFICIENS *(*DEF*) and *GLOBOSA *(*GLO*), *Arabidopsis APETALA3 *(*AP3*), *PISTILLATA *(*PI*), *AG *and embryogenetic *AGL15 *[25,36,40-42]. Positive or negative feedback loops in autoregulation maintain their expression in the development of floral organs or embryos after the initial induction. In tomatoes, *TOMATO AGAMOUS-LIKE 1 *(*TAGL1*) is possibly autoregulated [43]. The autoregulation of *RIN *might help to maintain its sufficiently high expression in ripening fruit, and consequently effectively regulate the expressions of direct target genes involved in the ripening process.

We also demonstrated here the direct interaction of RIN with the promoters of the genes involved in ethylene synthesis, *LeACS2 *and *LeACS4*, *in vivo *by ChIP. In the ethylene biosynthetic pathway, the conversion of S-adenosyl-L-methionine (SAM) to ACC by ACC synthase is a known rate-limiting step [44]. *LeACS2 *and *LeACS4 *regulate a massive increase in ethylene production of fruit associated with ripening, which is defined as system 2 ethylene production in contrast to system 1, which produces ethylene constitutively at a low level within pre-ripening fruits [7-9]. Recently, Yokotani et al. [9] demonstrated that ethylene production during ripening (system 2) consists of both autocatalytic and ethylene-independent systems and that transition from system 1 to system 2 occurs mainly via limited expression of *LeACS2 *and *LeACS4*, both of which are controlled by ethylene-independent developmental factors. Our finding strongly suggests that RIN directly regulates *LeACS4 *as well as *LeACS2*, and this finding is consistent with those of previous reports [6,8,9]. Meanwhile, direct regulation of *LeACO1 *and *ETR3 *expression by RIN cannot be confirmed at present due to relatively lower enrichment levels of the CArG boxes in their promoters in this study. In addition, a HD-Zip homeobox protein LeHB-1 directly regulates the expression of *LeACO1 *during fruit ripening [45], suggesting that RIN may not be involved in the direct regulation of *LeACO1*. However, we cannot exclude the possibility that RIN regulates the expression by binding to CArG boxes in the transposable element-like sequence in the *LeACO1 *promoter that could not be examined in this study. Regardless, our results certainly suggest that RIN contributes to the initiation of ethylene production at the onset of ripening through upregulation of *LeACS2 *and *LeACS4*.

We also demonstrated here the direct interaction of RIN with the promoters of ripening-induced genes, *PG*, *TBG4*, *LeEXP1 *and *LeMAN4*, *in vivo *using ChIP. This finding strongly suggests the direct transcriptional regulation of these genes by RIN, similar to its regulation of *LeACS2 *and *LeACS4*. Previous studies have shown that the expression of *PG *in tomato fruit is regulated by ethylene and that the promoter contains *cis *sites highly similar to the ethylene-responsive regions of the *E8 *and *E4 *genes [46-48]. However, Oeller et al. [49] previously revealed that *PG *expression during ripening is induced in an ethylene-independent manner, consistent with our view that RIN directly regulates the *PG *expression. These facts suggest that both pathways (ethylene and RIN) are effective in controlling the *PG *expression during ripening. Furthermore, previous analyses with suppression or overexpression of the four genes revealed that these genes are involved in cell wall modification but not enough to independently soften fruit [10-12,15,16,19,50,51]. These facts and our results therefore suggest that RIN induces expression of the genes involved in cell wall modification simultaneously during ripening, enabling the gene products to soften fruit in a cooperative manner.

### ChIP revealed novel RIN-binding CArG-box sites and a possible RIN-regulating gene

We successfully identified four novel RIN-binding CArG-box sites from ripening tomato fruit by ChIP. This led to the detection of the genes near the CArG boxes. Among these genes, SGN-U571769 is possibly regulated by RIN during ripening due to the *RIN*-associated expression pattern. This gene encodes a protein similar to Trx y1, which serves as an oxidoreductase and is an activator of plastidal peroxiredoxin Q (Prx Q) [52]. Prx Q plays a role in peroxide detoxification, redox signaling and defense against pathogens [53,54]. When it decomposes peroxides, Trx y1 is used as an electron donor [55]. From these observations, we assume that RIN participates in these phenomena in ripening fruit via activation of *Trx y1 *expression. However, the involvement of Trx y1 in the ripening process needs further clarification because no functional evidence of such involvement or of the apparently irregular position of CArG-box 133R as a *cis*-acting element has been reported. More extensive ChIP-based analysis will lead to comprehensive identification of the target genes of RIN and will thus help clarify the transcriptional regulation of gene expression for fruit ripening.

## Conclusions

Ripening of climacteric fruits is genetically controlled by intricate transcriptional cascades through ethylene and non-ethylene-mediated regulation. In this study, parts of the cascades involving RIN are revealed.

qChIP-PCR analysis of ripening tomato fruit and gel retardation assay demonstrated that RIN binds to the CArG boxes in the promoters of the genes involved in cell-wall modification (*PG*, *LeEXP1*, *TBG4 *and *LeMAN4*) and system-2 ethylene biosynthesis (*LeACS2 *and *LeACS4*) *in vivo *and *in vitro*. This suggests that RIN controls fruit softening and ethylene production by the direct transcriptional regulation of the cell-wall-modifying genes and *ACS *genes, respectively. The control of ethylene production by RIN during ripening means that RIN is also indirectly responsible for ethylene-inducible ripening processes together with an ethylene-mediated control. In addition, the presence of autoregulation of *RIN *itself is also suggested in this study, and thus explains how a rapid increase in the RIN transcription level occurs at the onset of fruit ripening.

Sequence analysis of the IPed DNAs from ripening tomato fruit, qChIP-PCR analysis and gel retardation assay revealed four new CArG-box sites bound by RIN. Of these four sites, the site 133R has a gene SGN-U571769 that encodes Trx y1 and shows a *RIN*-dependent expression pattern, suggesting that it is possibly regulated by RIN.

A series of binding analyses of RIN revealed that RIN can bind to a wider variety of CArG boxes *in vivo *than the consensus sequence previously determined *in vitro*. This implies the presence of other factors necessary for RIN to target fruit-ripening specific genes.

## Methods

### Chromatin immunoprecipitation

ChIP experiments were performed as previously described [6] using ripening tomato fruit at the pink coloring stage (4 days after breaker stage) where the expression of *RIN *is strongly induced. The immunoprecipitated (IPed) DNA fragments with anti-RIN antibody were recovered and purified. The resulting IPed DNA pool was used for qChIP-PCR as a template as described later.

The IPed DNA was also partially analyzed by sequencing and mapping as previously described [56]. In brief, both ends of the IPed DNAs were blunted with DNA polymerase I and phosphorylated with T4 kinase (Takara BKL kit; Takara Biotech, Otsu, Japan), followed by the addition of adenine (A) at the 3' end with *Taq *DNA polymerase (Ex*Taq*, Takara Biotech) and by the ligation of an adaptor (prepared by annealing of two oligonucleotides, L: 5'-AGCACTCTCCAGCCTCTCACCGAGT-3' and S: 5'-CTCGGTGAGAGG-3'). The resulting DNA fragments were amplified by PCR with the L oligonucleotide as a primer. The PCR products were size-fractionated by agarose gel electrophoresis into 0.2-1 kb and cloned into a pBlueScriptII SK(-) vector that was digested with *EcoR*V and supplemented in advance with thymine (T) at the 3'end, followed by the introduction into *Escherichia coli *(JM109 strain) cells. The cloned DNA fragments were sequenced with a BigDye Terminator cycle sequencing kit v3.1 and analyzed by an ABI Prism 310 genetic analyzer (Applied Biosystems, Foster City, CA).

### *In silico *motif search

Nucleotide sequences of the representative ripening-induced gene promoters were obtained from public databases: *LeACS2 *(accession no. X59139), *LeACS4 *(M88487), *LeACO1 *(X58273), *ETR *(*NR*, AY600437), *PG *(X14074), *TBG4 *(AF020390), *LeEXP1 *(U82123), *LeMAN4 *(AY046588), *PSY1 *(EF157835), *INV *(Z12027) and *RIN *(AF448522). Because the 5' region upstream of the protein-coding regions of *PSY1*, *PG*, *TBG4*, *LeEXP1*, *LeMAN4 *and *RIN *is less than 2 kb, their promoter sequences corresponding to 2 kb were complemented with Whole Genome Shotgun (WGS) data using the BLASTN program [57]. CArG boxes in the promoters were searched using the FUZZNUC program included in the EMBOSS package [58]. Sequence information for the promoters and motifs is summarized in Table 2.

The sequenced IPed DNA clones were mapped *in silico *on a draft genome sequence (WGS) of tomato released by the International Tomato Genome Sequencing Consortium (http://solgenomics.net/about/tomato_sequencing.pl) using BLASTN [57]. Using FUZZNUC [58], CArG-box motif sequences in the mapped regions were searched against the genomic sequences corresponding to the cloned IPed DNAs or the 1-kb genomic regions that mostly covered the clones when they were not fully sequenced.

### Enrichment test for *in vivo *binding analysis of RIN

Enrichment levels of the CArG boxes by ChIP were monitored using quantitative real-time PCR (qChIP-PCR) as described below. The IPed DNA pool was used for qChIP-PCR as a template. The pre-immune serum (without anti-RIN antibody; PI)-treated chromatin DNA pools and the total input chromatin DNA (without ChIP treatment) pools were used as templates for the negative and standard controls, respectively. The nucleotide sequences of the oligonucleotide primers specific to the respective CArG-box sites used in this study are listed in Additional File 1.

Quantitative ChIP-PCR analyses were performed using Power SYBR green PCR master mix and a 7300 real-time PCR system (Applied Biosystems) according to the manufacturer's instructions. In a 50-μl reaction mixture, 2 μl of the IPed DNA, the PI-treated or the input DNA pool was applied as a template. The PCR conditions were as follows: 95°C for 10 min, 40 cycles of 95°C for 10 sec, and 60°C (or 57°C depending on the primers; see Additional File 1) for 1 min, followed by a dissociation step. The measurements [quantification cycle (Cq) values] for the CArG boxes were normalized with those for the *Actin *gene, which is non-bound by RIN and thereby used as a reference [6]. The enrichment levels were represented as fold changes relative to the input DNA. An outline/checklist for qChIP-PCR has been generated based on a template provided in [59] (Additional File 3).

### Gel retardation assay

Gel retardation assays were performed as previously described [6] with a small modification. In brief, the DNA fragments including the normal or mutated CArG boxes (Table 1) were cloned. The mutated sequences were designed by replacing the first C and last G bases of the normal CArG boxes to T and A, respectively. The cloned DNAs were labeled by amplification with fluorescein isothiocyanate (FITC)-conjugated primers, purified and bound *in vitro *with RIN-MIK protein generated using a TnT SP6 quick-coupled transcription/translation system (Promega, Madison, WI) [6]. This protein, which lacks the C-terminal domain, was used because a clearer signal compared with that using the entire RIN protein could be generated [6]. The protein-DNA complexes were electrophoresed using polyacrylamide gel electrophoresis and detected using a typhoon 8600 (GE Healthcare Bio Science, Buckinghamshire, England) as previously reported [6].

### Gene prediction

A sequence similarity search for the genomic ~5 kb flanking the CArG boxes enriched by ChIP was carried out against a tomato expressed sequence tag (EST) database provided by Solanaceae Genomics Network (SGN unigene set; available at ftp://ftp.sgn.cornell.edu/unigene_builds/) using the BLASTN program [57]. The ESTs with the highest sequence similarity (at least >100 bp alignment length and >90% identity) to the respective genomic sequences were adopted. A sequence similarity search for these ESTs was performed against the non-redundant (nr) protein database at the National Center for Biotechnology Information (NCBI) using the BLASTX program [57]. Gene structures were predicted using the sim4 [60] and the FGENESH 2.6 [61] programs with a parameter for the tomato genome (available at the Softberry site: http://linux1.softberry.com/berry.phtml)

### Gene expression analysis

Expression levels of the genes were analyzed by quantitative real-time reverse transcription PCR (qRT-PCR) using oligonucleotide primers specific to each EST or *RIN *(Additional File 2). Using an RNeasy Plus Mini Kit (Qiagen, Hilden, Germany), total RNA was extracted and purified from tomato fruits of a normal (a genotype of *RIN*/*RIN*) plant at different stages (mature green, pink coloring and red ripe) and of a *rin *mutant (*rin*/*rin*) plant at periods corresponding to these stages, as previously described [29]. Complementary DNA was synthesized from total RNA using a PrimeScript II first cDNA strand synthesis kit (Takara Biotech) and then applied in real-time PCR as a template.

Quantitative RT-PCR analyses were performed in the same manner as for qChIP-PCR except that 2 μl of cDNA synthesis reaction mixture was applied as a template instead. Copy numbers of the objective transcripts were calculated from the measurements (Cq) using standard curves generated from a series of diluted PCR products for the respective genes. The data were normalized with that of a gene encoding clathrin adaptor complexes medium subunit (*CAC*; SGN-U314153) as a reference [62] (Additional File 2). An outline/checklist for qRT-PCR has been generated based on a template provided in [59] (Additional File 4).

## Authors' contributions

MF and YI conceived the study and designed all the experiments. TN grew and prepared the tomato fruits. MF performed all the analyses. MF and YI interpreted the experimental data and participated in writing the manuscript. All the authors have read and approved the final manuscript.

## Supplementary Material

Additional file 1 **Primer pairs specific to the CArG-box sites used for enrichment test**. Primer pairs used in this study. The melting temperature (Tm) of each pair for qChIP-PCR is indicated in the right column. Click here for file

Additional file 2 **Primer pairs specific to the tomato ESTs found in this study used for expression analysis and results of similarity searches for the ESTs**. Primer pairs used in this study. Known proteins with the most significant similarity to proteins encoded by the ESTs are shown. Click here for file

Additional file 3 **A checklist outlining the DNA to qChIP-PCR quality/methodology as described in **[59]. Click here for file

Additional file 4 **A checklist outlining the RNA to qRT-PCR quality/methodology as described in **[59]. Click here for file
